# Multidisciplinary approach as a treatment option for abdominal wall reconstruction in patients with heart failure: A case report

**DOI:** 10.1016/j.ijscr.2022.107770

**Published:** 2022-10-24

**Authors:** María Paula Gamboa Bernal, Sebastián David Contreras, Alejandro Gonzalez, Paulo A. Cabrera Rivera, Carlos J. Perez

**Affiliations:** a General Surgery Research Group, Fundación Cardioinfantil-La Cardio, Bogotá, Colombia; b Universidad de La Sabana, School of Medicine, Chia, Colombia

**Keywords:** IH, Incisional hernia, HF, Heart failure, LVEF, Left ventricle ejection fraction, CeDAR, The Carolinas Equation for Determining Associated Risk, Case report, Abdominal hernia, Multidisciplinary approach

## Abstract

**Introduction and importance:**

Incisional hernias are among the most frequent complications of abdominal surgery, with an incidence of 4–10 % of patients [1]. The multidisciplinary approach according to the patient's needs and their comorbidities has been shown to improve postoperative outcomes. This case report highlights the importance of a multidisciplinary approach including cardiology, general surgery, plastic surgery anesthesiology and intensive care unit for abdominal wall reconstruction in a patient with heart failure and reduced ejection fraction.

**Clinical presentation:**

We present a case of a 61-year-old patient with long-standing incisional hernia, without surgical correction due to the patient's condition and multiple comorbidities, advanced heart failure with reduced left ejection fraction (10–15 %) who underwent a multidisciplinary approach by cardiology, plastic surgery, anesthesiology, intensive care unit, and general surgery.

**Discussion:**

The patient underwent abdominal wall reconstruction without complications. Due to multiple comorbidities, the patient was admitted in the ICU in the immediate postoperative period. He was discharged 9 days after surgery. The patient did not report long-term complications.

**Conclusion:**

Heart failure is associated with an increased risk of cardiovascular complications during surgical hospitalization. In patients with multiple comorbidities, the multidisciplinary approach represents an essential strategy in order to improve the surgical outcome, reduce costs to the health care system, and improve the patient's quality of life.

## Highlights

• Heart failure is associated with an increased risk of complications after non-cardiac surgery during surgical hospitalization
• In patients with multiple comorbidities, the multidisciplinary approach represents an essential strategy
• Standardized multidisciplinary programs are required in those who will be taken to abdominal wall reconstruction

## 1 Introduction

Incisional hernias (IH) are defined as “defects in the abdominal wall around postoperative scars, perceptible or palpable by clinical examination or by imaging studies” [1]. IH are one of the most frequent complications of abdominal surgery, with an incidence of 4–10 % of patients after abdominal surgery. The frequency of presentation increases to 30–50 % in patients with seromas, hematomas or surgical wound infection [1]. The surgical technique used for abdominal wall closure in patients with risk factors such as age, comorbidities such as nutritional status, diabetes mellitus, obesity, smoking, chronic use of steroids and states of immunosuppression of the patient; can significantly influence the incidence of incisional hernia [1]. Patients may experience unspecific symptoms such as abdominal pain and gastrointestinal problems. If the hernia follows its natural course and remains untreated the most severe complication is incarceration and strangulation, which increases mortality and morbidity [2], [3].

Heart failure (HF) is recognized to be associated with an increased risk of complications after non-cardiac surgery, including cardiovascular complications during surgical hospitalization such as perioperative acute myocardial infarction (MI), ischemic stroke, acute pulmonary embolism, and cardiac arrest. Moreover, HF represents an additional risk factor that increases anesthesia-related cardiovascular complications. We present a case of a patient with a history of advanced heart failure with reduced left ventricle ejection fraction (LVEF) who underwent multidisciplinary approach to an abdominal wall reconstruction in a fourth-level health care center [4]. This case report has been reported according to the SCARE Criteria [5].

## 2 Case report

**Fig. 1:**
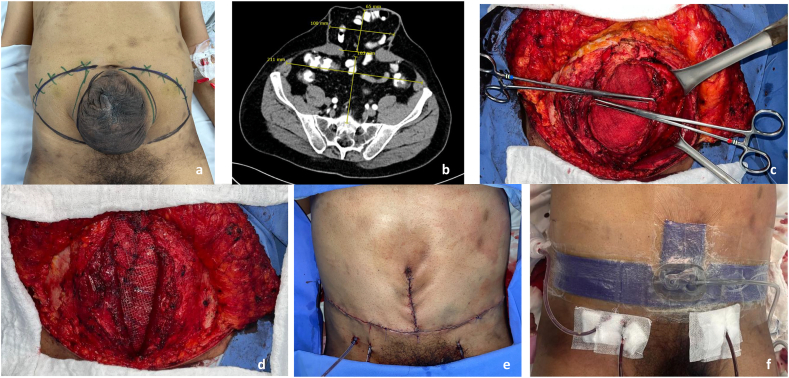
Trans-operative record of the abdominal wall reconstruction. a) Clinical presentation of the patient showing the abdominal incisional hernia. b) Preoperative Axial Computed Tomography. c) Intraoperative finding consisting of adequate intestinal perfusion, with no signs of necrosis or suffering. The adhesions and the multiple sub-eventrations are also visible. d) Polypropylene mesh suitably positioned. e) Immediate postoperative result after abdominal wall reconstruction. f) Placement of a sub atmospheric pressure system.

A 61-year-old male patient presented to the emergency room with signs of decompensated HF due to dyspnea, lower limb edema, non-anginal chest pain and long term diffuse abdominal pain. Past medical history revealed advanced heart failure AHA stage D of probable alcoholic etiology with reduced LVEF of 10–15 %, severe functional mitral insufficiency, sudden death recovered with implantable cardioverter- defibrillator (ICD), chronic kidney disease, chronic obstructive pulmonary disease (COPD), and abdominal hernia secondary to open appendectomy. Physical examination showed an incisional hernia classified as M3-M5W2 according to the European Hernia Society (EHS) classification, without abdominal pain and signs of strangulation or incarceration (Fig. 1a) and clinical findings consistent with decompensated HF.

However, due to the multiple comorbidities of the patient, it was considered a priority to treat the underlying disease before evaluating a possible surgical intervention. The patient had been treated as an outpatient with cycles of intermittent inotropy with levosimendan. Initially the patient was evaluated by cardiologists, who considered he had a new decompensation of his HF classified as Stevenson B due to the hemodynamic pattern. He was treated with furosemide, with partial response to treatment. For that reason, the furosemide dose was increased and dual angiotensin receptor and neprilysin inhibitor was adjusted.

Once the patient was treated for the decompensated HF, cardiology raised the possibility of multidisciplinary management for abdominal wall reconstruction due to impact of the patient's quality of life. Thus, the patient was evaluated by a general surgeon with special training in abdominal wall repair, who ordered a computed tomography scan (CT scan) to evaluate the dimensions of the hernia defect and rule out loss of domain. Additionally, the Carolinas Equation for Determining Associated Risk (CeDAR) risk was calculated in 23 %, a significant risk of postoperative and wound complications, and high cost in-hospital charges.

The patient was taken to an abdominal CT scan that showed an abdominal wall defect that compromised the umbilical zone to suprapubic zone, associated with diastasis of the rectus abdominis of 70 mm, the hernia sac measured 60 × 96 mm in the axial plane and 145 mm in the craniocaudal plane (Fig. 1b). With the images and the measures, the Tanaka index was calculated at 0.06, so loss of abdominal domain was ruled out, and the patient was considered a candidate for abdominal wall reconstruction, without requiring preoperative therapies to prevent abdominal hypertension (for example, preoperative progressive pneumoperitoneum or botulinum toxin injection).

The patient was assessed by anesthesiologists who catalogued him as an ASA (American Society of Anesthesiologists) III and considered that he had high risk of perioperative complications but could be taken to surgery with postoperative surgical surveillance in the intensive care unit (ICU). Additionally, the patient was evaluated by plastic surgery for collaborative surgical management in closure the skin defect coverage.

The surgery was led and performed by a general surgeon with special training in abdominal wall repair. Under general anesthesia, an incision was made around the previous laparotomy scar and mark made by plastic surgery. An 8 cm composite local skin flap stalk was started in all directions until the healthy fascia was identified and the herniated sac was opened. Adequate integrity of the small and large bowels was assured. All adhesions were released (Fig. 1c).

Afterwards, the Rives-Stoppa technique was used to reconstruct the abdominal wall. The entire sac was resected; a longitudinal incision was made out on the posterior aponeurosis of the abdominal rectus muscles. A retromuscular plane was dissected underneath the rectus abdominis muscle. Then, primary closure was performed without tension of the posterior aponeurosis of the abdominal rectus muscles. Posteriorly, a 40 × 40 medium density polypropylene mesh was placed above the posterior aponeurosis of the rectus abdominis muscle, covered by the anterior rectus sheath, a silicone drain was placed above the mesh (Fig. 1d). Another drain was placed anterior to the fascia of the rectus abdominis muscle, in the dissection spaces of the skin flaps. After finishing the intervention by general surgery, plastic surgery intervened on the patient by closing the skin and subcutaneous cellular tissue (Fig. 1e), placing a sub atmospheric incisional pressure system (Fig. 1f).

The patient was treated in the ICU in the immediate postoperative period. He presented postoperative dyspnea that was treated with oxygen via nasal cannula. A bedside chest radiography showed pulmonary edema. For this reason, the patient was treated as a decompensated heart failure with furosemide. The patient showed a satisfactory in-hospital evolution and was discharged 9 days after surgery. 7 days after the surgical intervention, the patient attended a postoperative control appointment, showing wounds in adequate condition, with no evidence of hernia recurrence.

## 3 Discussion

Heart failure (HF) is associated with an increased risk of cardiovascular complications during surgical hospitalization. According to Smilowitz et al. patients with diagnostic of HF undergoing non cardiovascular surgical procedures had a greater mortality compared to those without HF (4.8 % vs. 0.78 % P < 0.001; adjusted odds ratio (aOR) 2.15, 95 % confidence interval (CI) 2.09–2.22) [6].

Our institution is a fourth-level health care center; thus, we have different medical specialties, and we treat patients with multiple comorbidities; therefore, the management of abdominal wall reconstruction is carried out in a multidisciplinary approach, according to the patient's requirements. Complex abdominal wall reconstruction continues to challenge multidisciplinary surgical teams who must address the structural, functional and aesthetic components unique to each patient in order to improve surgical outcomes, thus, interdisciplinary collaboration is essential [7]. Furthermore, determining the Tanaka index represents a fundamental strategy in patients with abdominal wall hernia because it allows defining whether there is loss of domain, which changes the surgical approach [3].

In addition, in patients with multiple comorbidities, collaborative work between the different medical specialties and preoperative care is essential to bring the patient to the operating room in the best possible conditions and improve postoperative outcomes. The presence of modifiable risk factors such as obesity, diabetes, and smoking, can represent an opportunity for preoperative optimization, improving surgical outcomes and reducing postoperative complications [7], [8]. Identifying preoperative risks allows surgeons to calculate and discuss predicted complication risks, which are associated with preoperative modifiable comorbidities. Current risk calculation models applicable to hernias include the Carolinas Equation for Determining Associated Risks (CeDAR), an application that utilizes patient risk factors to predict postoperative cost and risk of wound complication [8], [9]. Moreover, abdominal wall reconstruction surgeries can present various postoperative complications such as recurrence, wound infection, wound dehiscence, among others, which can increase the morbidity of the surgical procedure [1], [3].

In this case report, the patient had multiple comorbidities, including heart failure with reduced LVEF, this conditioned a great perioperative and postoperative risk, for this reason, he was managed in a multidisciplinary approach with cardiology, anesthesiology, plastic surgery and general surgery. The patient was taken to surgery once his comorbidities were compensated, and the requirements requested by each of the services for postoperative management were met. In our experience, the multidisciplinary approach to patients undergoing abdominal wall reconstruction surgery represents a fundamental strategy to achieve compensation of comorbidities, reduce the patient's operative risk, reduce patient morbidity and mortality, reduce hospital stay and health care system costs, and improve the patient's quality of life.

## 4 Conclusion

Multidisciplinary management in patients undergoing abdominal wall reconstruction represents an essential strategy, especially in those patients with multiple comorbidities and advanced chronic diseases such as heart failure. Further studies and evidence are required to achieve strong clinical recommendations and apply evidence-based medicine. Additionally, more standardized multidisciplinary programs aimed at the integral management of patients with comorbidities are required in those who will be taken to abdominal wall reconstruction, in order to have a specific handling route for the approach.

## Ethical approval

This case report was submitted and approved by the ethics committee of Fundación Cardioinfantil- Instituto de Cardiología.

## Funding

This research did not receive any specific grant from funding agencies in the public, commercial, or not-for-profit sectors.

## CRediT authorship contribution statement

María Paula Gamboa Bernal: Conceptualization, Investigation, Writing- Original Draft and Visualization

Sebastián David Contreras: Conceptualization, Investigation, Writing- Original Draft and Visualization

Alejandro Gonzalez: Supervision, Writing – Reviewing and Editing, Validation

Paulo A. Cabrera Rivera: Supervision, Writing – Reviewing and Editing, Validation and Project Administration.

Carlos J. Pérez: Supervision, Validation

## Guarantor

Paulo A. Cabrera MD, MSc.

## Research registration

None.

## Consent for publication

Written informed consent was obtained from the patient for publication of this case report and accompanying images. A copy of the written consent is available for review by the Editor-in-Chief of this journal on request.

## Provenance and peer review

Not commissioned, externally peer-reviewed.

## Declaration of competing interest

The authors certify that they have no affiliations with or involvement in any organization or entity with any financial interest in the subject matter or materials discussed in this manuscript.
